# The Electrome of a Parasitic Plant in a Putative State of Attention Increases the Energy of Low Band Frequency Waves: A Comparative Study with Neural Systems

**DOI:** 10.3390/plants12102005

**Published:** 2023-05-16

**Authors:** André Geremia Parise, Thiago Francisco de Carvalho Oliveira, Marc-Williams Debono, Gustavo Maia Souza

**Affiliations:** 1 School of Biological Sciences, University of Reading, Reading RG6 6AH, UK; 2 Laboratory of Plant Cognition and Electrophysiology (LACEV), Department of Botany, Institute of Biology, Federal University of Pelotas, Capão do Leão 96160-000, RS, Brazil; fthicar@gmail.com (T.F.d.C.O.);; 3 PSA Research Group, 91120 Palaiseau, France

**Keywords:** selective attention, low waves, gamma waves, parasitic plants, EPG activity, plant cognition, plant-plant interaction, plant electrophysiology, average band power

## Abstract

Selective attention is an important cognitive phenomenon that allows organisms to flexibly engage with certain environmental cues or activities while ignoring others, permitting optimal behaviour. It has been proposed that selective attention can be present in many different animal species and, more recently, in plants. The phenomenon of attention in plants would be reflected in its electrophysiological activity, possibly being observable through electrophytographic (EPG) techniques. Former EPG time series obtained from the parasitic plant *Cuscuta racemosa* in a putative state of attention towards two different potential hosts, the suitable bean (*Phaseolus vulgaris*) and the unsuitable wheat (*Triticum aestivum*), were revisited. Here, we investigated the potential existence of different band frequencies (including low, delta, theta, mu, alpha, beta, and gamma waves) using a protocol adapted from neuroscientific research. Average band power (ABP) was used to analyse the energy distribution of each band frequency in the EPG signals, and time dispersion analysis of features (TDAF) was used to explore the variations in the energy of each band. Our findings indicated that most band waves were centred in the lower frequencies. We also observed that *C. racemosa* invested more energy in these low-frequency waves when suitable hosts were present. However, we also noted peaks of energy investment in all the band frequencies, which may be linked to extremely low oscillatory electrical signals in the entire tissue. Overall, the presence of suitable hosts induced a higher energy power, which supports the hypothesis of attention in plants. We further discuss and compare our results with generic neural systems.

## 1. Introduction

To survive in a world where the parameters change constantly, organisms must keep track of these variations. This requires an active engagement with the cues that can be perceived by the sensory organs or surfaces and that will cause rearrangements in the internal structure of the organism. Ultimately, this leads to adjustments in the behaviour of the organism in relation to the perceived cues to keep its homeostasis within an acceptable range [1,2,3]. However, there are limits to both the capacity to sustain those engagements, and the behaviours that are possible to entail. To balance this out, attention is required.

Attention is the cognitive phenomenon that mediates the interaction between the internal states of an organism and the features of the environment with which the organism will actively engage [1,4,5]. Despite the focus on external stimuli, attention can be directed to internal stimuli as well (e.g., attention towards an organ or limb that is aching, working memory, or introspective thoughts in humans) [6]. Attention is a dynamic process that is constantly altered as both internal and external states change. In humans, a whole taxonomy of attention types has been recognised [5,6], but basal forms of attention should be observed in any organism that must make trade-offs between its needs and the many possibilities of engagements (or affordances, in Gibsonian parlance) the environment offers [7,8]. In this case, the most basal form of attention, *selective attention*, is probably shared by many different taxa [8,9]. Here, we discuss the possibility of plants being attentive.

Despite the structural simplicity of their bodies, plants have very complex physiologies, and many channels for engaging with the world. Plants have a panoply of senses that constantly monitor environmental variables [10,11]. All these senses are distributed by virtually all their bodies, and not concentrated in sense organs as often happens with animals. Consequently, plants are subjected to what could be an overwhelming number of stimuli, and responding reliably to those relevant for immediate and future survival is of the utmost importance. How, then, could plants perceive, process, and use all the information the environment potentially offers?

Part of the issue is solved by the modular constitution of plants, where the modules act semi-independently upon the stimuli that are relevant to them in their contexts, without requiring much interaction with the other modules. However, sometimes, plants face challenges, or perform actions that require the coordination of many or even all of the modules. Examples include resisting to abiotic stresses such as drought or excessive temperatures, growing away from potential competitors, growing or orientating leaves towards light sources even when light is absent [12,13,14,15,16], and movements towards structural supports in the case of climbing plants [17,18,19]. These behaviours necessarily require focusing on the most relevant stimuli to the task in hand and ignoring the stimuli irrelevant or not related to the task. In other words, they require attention [20].

In the case of plants, attention is likely observable using electrophytograms (EPGs) in the form of spontaneous bioelectrical variations of electrical potential. These potential variations usually operate in the microvolt range and are continuously produced by presumably all plant species [21,22]. These bioelectrical signals would be modified when plants perceive environmental signals or cues and prioritise one or some of these signals and cues over others [23,24,25]. The effect of these interactions on the electrophysiology of plants has been observed in different plant species under different conditions [26,27,28,29,30,31,32].

It might feel odd to discuss about attention in plants, as this phenomenon is traditionally studied in humans or animals phylogenetically very close to us. However, attention is an evolved characteristic, and hence, if it is present in humans and our evolutive close relatives, it should therefore be present in our common ancestors in some form. How far in the tree of life we can find this phenomenon remains to be elucidated. However, the fact that attention connects internal physiological states with environmental information to guide behaviour hints to a rather ancient origin, and attention could therefore be present in a range of organisms far wider than expected. Indeed, recent studies demonstrate attention not only in mammals but also in birds, fish, and invertebrates [33,34,35,36]. Other studies also pointed to correlations between the basal attentive system of insects and those of humans [8,37,38]. However, attention is not necessarily restricted to the animal kingdom.

Marder [20] attempted to define attention in non-zoocentric terms, so that hypotheses focusing on attention do not a priori reject non-traditional groups of organisms from their frameworks. In defining attention as a disproportionate investment of energy of a cell, tissue, or organism into a particular activity, or in the reception of a stimulus or set of stimuli, Marder was the first to propose that attention could be a phenomenon observed in plants [20]. A few years later, when studying the electrophysiology of the parasitic dodder plant (*Cuscuta racemosa*), Parise et al. [31] found what they identified as the first empirical evidence of a state of attention in a plant when *C. racemosa* twigs were near to their hosts. A hypothesis of plant attention was then further developed to combine the theory and practice in a framework of attention that would make sense to the biology of plants and other sessile organisms [9].

When reviewing the literature on plant electrophysiology, Parise et al. [9] observed that attention in plants could follow the same pattern in different species. In short, attention could be observed in plants through EPG analyses when the complexity of the electrome (meaning the collection of all the electrical activity of an organ or tissue) drops while the correlation between the signals increases. Following the definition of Marder [20], typically, an increase in the energy of the signals would also be measured. This happens because, when involved in an activity or receiving stimuli that likely requires attention by the whole plant, the modules would synchronise their bioelectrical behaviour to coordinate their actions in response to the stimuli. This behaviour was observed in soybean (*Glycine max*), common bean (*Phaseolus vulgaris*), and dodder plants (*Cuscuta racemosa*) [9]. Another recent study on *P. vulgaris* analysed the electrome of local leaves (i.e., leaves that received heat and/or wounding stimuli) and systemic leaves (i.e., leaves that did not). In both the local and systemic leaves, the electromes presented a transiently decreased complexity accompanied with an increased correlation [39], thereby supporting the hypothesis that attention in these cases requires the synchronisation of all the modules. Putative attention in plants most likely leads to the generation of specific EPG patterns and electromic signatures, which are a phenomenon greatly facilitated by the mesological plasticity of plants in their singular milieu [40].

The studies mentioned above corroborate the hypothesis of plant attention as formulated by Parise et al. [9] and suggest that electrophysiological analyses are valuable to empirically observe this phenomenon. However, the analyses proposed by Parise et al. [9] could be further improved to better reflect the presumed attentional dynamics. For example, such as in humans and non-human animals, plants could also have different ranges of wave frequencies related to different stimuli or activities in a form analogous to the brain waves (e.g., alpha, theta, gamma, and delta waves). Understanding how the energy of the signals is distributed across the wave frequencies could provide important insights into the electrical ecophysiology of plants, and yield clues of how the mechanisms for information processing operate in aneural organisms. Therefore, analyses that better capture the subtleties of the electrical signals in plants under states of active perception or (putative) attention should be developed to convey more precise information on the dynamics of this cognitive ecophysiological phenomenon.

In this study, we revisited the electrophysiological data obtained from the dodder plant, *C. racemosa*, a holoparasitic plant, when presented to two different hosts: the bean plant, a suitable host; and the wheat plant, a host that dodders cannot parasitise [31]. We assumed that the plants were under a state of attention in the presence of their hosts as their electromes followed the dynamics described above and described in detail in Parise et al. [9]. Upon host perception, the electrome changed its dynamics as the parasitic plants would be focusing their attention mainly on the suitable hosts and organising their physiology to grow towards the hosts and change their pigment content according to the species detected (see [31] for details). In other words, when a meaningful cue appeared in the environment, the plants invested more energy in the perception of this cue and in the actions required to respond adaptively to it.

In the present study, we hypothesised that there would be a difference in the energy of the electrophysiological waves of the dodder electromes. This difference would probably be more evident in the lowest frequencies, as according to how plant electrophysiological activity usually operates [22]. We employed a different and more reliable technique, time dispersion analysis of features (TDAF) to infer the increase of energy manifested through the difference of potential (DDP), and the average band power (ABP) to separate the electrophysiological activity of *C. racemosa* into their respective band frequencies.

## 2. Results

**Figure 1:**
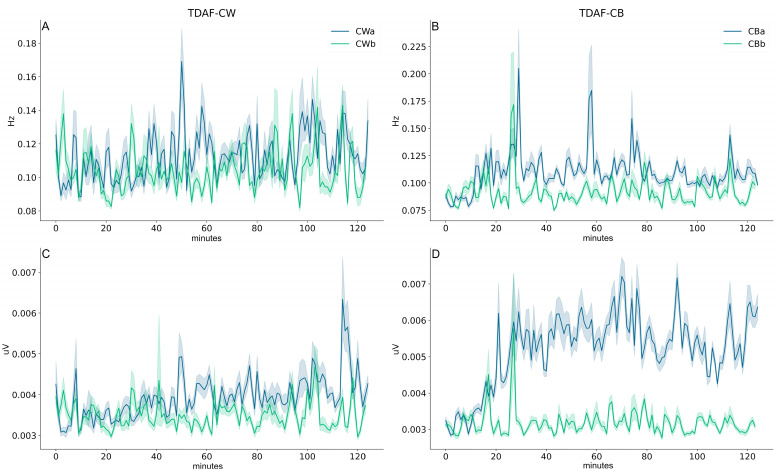
In (**A**,**B**), the mean of the frequencies of all the dodder plants analysed (*n* = 23) calculated for every minute are shown in the *Y* axis. (**C**,**D**) show the mean of the difference of potential of all the plants. The *X* axis shows the time of the recording in minutes. The data of dodders exposed to the wheat plants is displayed on the left side of the figure (**A**,**C**), and on the right side (**B**,**D**), the data of dodders exposed to the bean plants is shown. The darker lines indicate the median of the data, and the lower and upper limits of the shadow represent the minimal and maximum values obtained, respectively. The green line represents the values of the dodders before being exposed to the host, and the blue line, after exposure.

**Figure 2:**
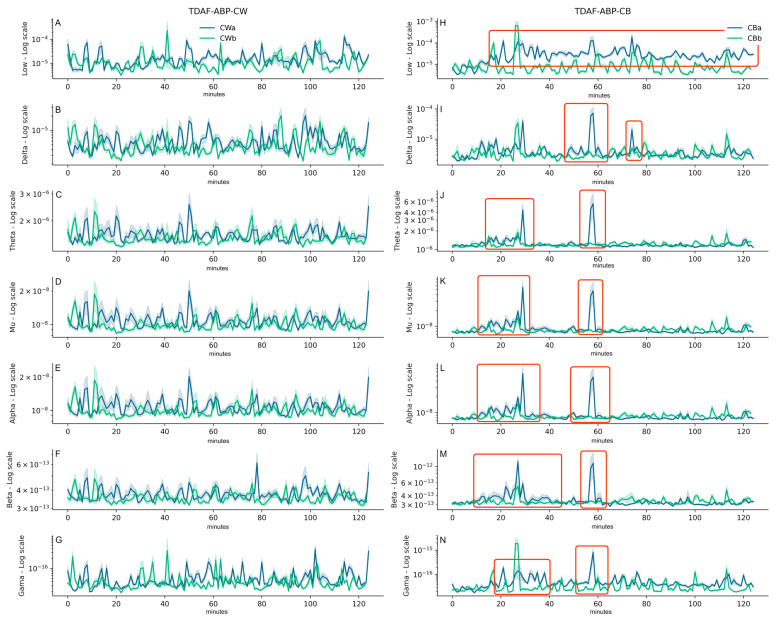
ABP analysis for the electrical signals of the dodders for all the plants studied (*n* = 23 per treatment). The ranges were named low (0.0–0.5 Hz; (**A**,**H**)), delta (0.5–4 Hz; (**B**,**I**)), theta (4–8 Hz; (**C**,**J**)), mu (9–11 Hz; (**D**,**K**)), alpha (8–13 Hz; (**E**,**L**)), beta (13–30 Hz; (**F**,**M**)), and gamma (20–100 Hz; (**G**,**N**)) waves. The *Y* axis shows the power values returned for each range of frequencies, and the *X* axis shows the time minute-by-minute during the recordings of the data (2 h before and 2 h after exposure to hosts). The darker lines indicate the median of the data, and the lower and upper limits of the shadow represent the minimal and maximum values obtained, respectively. The green line represents the values of the dodders before being exposed to the host, and the blue line, after the exposure. Relevant changes in the electrome dynamics were highlighted in red.

The results based on TDFA (time dispersion analysis of features) were obtained combining the data of all the dodder plants in each treatment, with the lines in Figure 1 representing the median for all the values. Therefore, the time series analysed in this study result from the analysis of the twenty-three individual time series of each plant in the same treatment. The shaded areas demarcate the range between the highest and lowest values obtained. The series shown in Figure 1 and Figure 2 show a general behaviour where, despite the higher dispersion around the median in several moments, overall, the power distribution was mostly uniform.

In Figure 1A,B, the mean of the frequencies decomposed by the fast Fourier transform (FFT) is shown at every minute during the entire duration of data recording (2 h). In Figure 1A, the mean of the frequencies before and after the dodder being exposed to wheat can be observed. In this case, there was no significant difference found in the general behaviour. Around 50 min post-presentation to the host, there was a spike observed, but the dispersion was also high in this moment, therefore suggesting that this effect could be due to one or a few plants that presented a different behaviour in this moment. However, when the dodders were exposed to the bean plant (Figure 1B), there was a clear increase in the frequencies observed around 20 min later, and this increase persisted until the end of the recordings. In this case, even though there was a high dispersion around the spikes, the median after exposure to the suitable host plant was kept at a higher level than before exposure throughout the entirety of the recording. Additionally, a clear micro-voltage increase and persistence in the difference of potential (DDP) after dodder exposure to the bean plants was observed (Figure 1D). However, this behaviour was not observed in dodders exposed to wheat plants (Figure 1C).

Figure 2 shows the ABP analyses for the signals sampled. Again, the left side of the figure shows the values of dodders presented to the wheat plants (Figure 2A–G), and on the right side, dodders presented to the bean plants (Figure 2H–N). The figure shows the band frequencies for low (0.0–0.5 Hz), delta (0.5–4 Hz), theta (4–8 Hz), mu (9–11 Hz), alpha (8–13 Hz), beta (13–30 Hz), and gamma (20–100 Hz) waves. The behaviour of the signals after the dodder being presented to the bean plant was quite distinct from before (Figure 2H–N, highlighted in red). After around 20 min, there was a sustained increase observed in the energy of the low waves throughout the recording (Figure 2H). The delta waves showed two peaks of energy investment, one at around 60 and the other around 80 min after exposure of the dodders to the bean plants (Figure 2I). In all the other band frequencies, there was a peak in energy investment observed at around 20 and 60 min after presentation to the host (Figure 2J–N)—except for gamma waves after 20 min, where the main peak occurred *before* presentation to the host. It was noticeable that the dispersion of the data was narrow, suggesting little variation in the behaviour of the plants that were analysed.

## 3. Discussion

Parise et al. [31] demonstrated that the bioelectrical activity of *C. racemosa* changes when the parasite is near a potential host, and that this change is not the same to both hosts. The results suggested that dodder plants recognise different host species from a distance, and this difference was observed already at the level of electrical signalling. The change in the dodders’ electrome was starker in the plants presented to the suitable host, the bean plant. Additionally, the dynamics of the electrome suggested that the dodder was being attentive to the cues of the host plants. Here, we investigated whether there is a difference in the investment of the electrome energy, and how this energy is distributed in the different band frequencies. We observed that dodders that were presented to the bean (i.e., the suitable host) invested more energy in their electromes than the dodders presented to the wheat plant when it comes to the electrical signals produced. This was evident not only in the increase of the DDP (Figure 1) but also in the distribution of frequencies given by the ABP analysis (Figure 2).

In this work, the same protocol for power distribution in band frequencies used in neuroscience was employed. This was necessary, as we are unaware of protocols specific for plants that permit the categorisation of the bands for different plant behaviours, as indicated by changes in the electrome. Consequently, we must have several caveats when interpreting the results. In neuroscience, each band typically demonstrates a specific behaviour or activity of the brain. For example, delta waves indicate a low cognitive activity [41], and gamma waves are related to learning and attention [42]. Since plants are very distinct beings, their bioelectrical behaviour was expected to be quite different, and likely more related to lower frequencies [43]. Accordingly, we found higher activity in this range of frequencies, here called low (0.0–0.5 Hz). However, spikes of activity were found in all band frequencies, mainly when the dodders were presented to the bean plants. When combining the ABP technique with TDAF, it was evident there was a change in the energy of the frequencies when the dodder is perceiving a salient feature of the environment.

Our results corroborate the hypothesis that plants under putative states of attention or active perception increase the energy expended in this activity [9]. This was particularly clear in this study when a suitable host was presented to *C. racemosa*. After approximated 20 min, the dodder allocated more energy to the electrome, specifically to low wave frequencies, and after around 60 min, a peak in the energy of the frequencies in all the plants was observed in the dodders presented to the bean plant.

Although it would be important to further investigate the links between the cyclic and low-frequency activities in *C. racemosa* and many other plants, our results indicated that, whatever the cognitive value we attribute to the perceptual process involved (such as selective attention), the operating mode was, in the particular case of the dodder: (1) host-selective (operational choice), (2) energy-intensive (in proportion to the frequencies detected), (3) associated with a possible state of attention on transdisciplinary grounds [9], and (4) of a mesological order (*Umwelt* of plants, a direct link with their singular milieu [44,45]).

This is in line with other studies on electrome complexity [26,40,43], including some that demonstrated specific electromic signatures in pathogen-infected plants [28,46], bioelectric patterns of oscillations recorded in loco in *Miconia* sp. [30], and bioelectrical reaction of fruit petioles when fruits are chewed by caterpillars [32]. Our study now shows that low-frequency peaks likely predominate in these electromes, but higher frequency bands also exist, which is a novel finding. To our knowledge, gamma-like waves in plants were never recorded before this study.

Frequency bands of EPGs are different and much slower in plants. However, they fall within the range of classical EEG activities [47,48,49] while being clearly different from them, as was demonstrated early on [23]. Specifically, they behave similarly in three main aspects:

(1) The complex functioning of ion channels and dipoles in cell membranes (apart from differences in the ions involved in plant and animal signals), whose direct influence on the shape of the waves has been confirmed at least in human brains with computer models [50]. How plant ion channels influence bioelectrical oscillation in plants remains to be elucidated.
(2) The generation of spontaneous low-voltage bioelectrical signals (background EPG activity) through layers of tissue dipoles that correspond for brainwaves to extracellular EEG ionic currents [51,52], and intracellular electromagnetic field potentials that can be recorded by MEG [53]. Spontaneous EPGs, as part of the electrome, may synchronise when plant tissues are stimulated [23,24,54,55].
(3) The nature of the signals collected in the plant tissues, ranging within the microvolt amplitude (5–250 μV) and relatively low frequencies (0.5–15 Hz)—despite being, as shown here, lower, and less diversified than in humans and non-human animals.

Nevertheless, the electrogenesis observed in plants is different from local field potentials (LFPs) or cerebral potentials observed in animals. These bioelectrical events reflect the algebraic sum of time-synchronised synaptic potentials from large populations of neurons, including interneuronal architecture and specialised brain networks that function as intracerebral generators and pacemaker systems [56]. They contribute to the permanent generation of frequency bands that are classically linked to states of vigilance, attention, or sleep in humans or non-human animals [57,58,59,60].

On the other hand, the plant’s perceptive system, including the spontaneous bioelectrical oscillations within plant bodies, involves ion channels that respond to chemical or physical stimuli [61]. As a result, there is a remarkable convergence in some plant and animal bioelectrical processes that suggest a deep evolutionary origin of the same mechanisms [24,45,62]. Plant intrinsic capacity to generate a permanent electromic activity is only now beginning to be explored [26,40]. There is an exciting potential for insights into the electrophysiological nature of plants and its relation to the establishment of scale-free states of self-organised criticality (SOC) to be made that can be quantified during ‘sensory hook-ups’ [26].

The electrome exhibits spontaneous and complex dynamics that is continuously generated and characterised by transition states. These transition states seem to underpin active perceptions and physiological modifications during complex tasks that require selective attention, memorisation, habituation, and associative learning [15,63,64], which require a cognitive apparatus [65].

The activity resulting from the electrome would be underpinned specifically by the dynamic non-linear organisation of cellular networks and bio-oscillators linked to certain tissue layers (e.g., meristems [66]). This is similar to the observed increase in the relative strength of certain brain rhythms at the neurobiological level, such as the theta frequency band of the parietal and temporal lobes during auditory stimulation [67], or during more complex phenomena such as binding or perceptual binding following visual stimulation. Different plant organs or modules also exhibit electromic activity in response to stimuli, similarly to how the brain exhibits increased activity in certain frequency bands following stimulation [24,54].

Temporal synchronisation of neural activity [68,69,70] involves the brain’s ability to synchronise the oscillatory phases of neurons from different regions to reconstruct both the shape and colour of an object or image. Similarly, our working hypothesis was that synchronising the electrical activity of different plant parts is the basis of plant electrome plasticity [26,39,40], and is one of the major keys to understand complex cognitive behaviours at the plant–environment interface. Spontaneous variations in the EPGs can indicate the real-time reaction of plants to stress and environmental stimuli in the form of spatiotemporal patterns that are linked to a specific stimulus or action. The oscillatory behaviours of plants, often involving Ca^2+^ waves, have been studied and modelled [55,66,71,72]. *C. racemosa* and many other plants have predominantly low frequency ranges related to energy expenditure that might increase during operational choice and attention facing external cues [9]. This phenomenon is remarkable and may have similarities with information processing at the neural scale [24,25,45,73,74].

Low-frequency cortical oscillations (<1 Hz), such as the K-complex, have been observed in the brain during sleep, and similar low-frequency EPG emissions have been observed in plants [25,45,75,76,77]. Although these processes cannot be compared directly, the mechanisms underlying the electromes of living organisms present similarities that could reflect a strong mesological plasticity linked to the biology and evolution of living species and their environment [25,45].

The aim now is to link these behaviours to cognitive typologies specific to plants (e.g., dynamic coupling mechanisms, electromagnetic and ecoplastic interfaces, distributed, extended, or embodied cognition, and learning), and to show more specifically how these putative states of selective attention could enable dodders to identify which prey to parasitise, and how much energy to allocate to this operation. This same type of behaviour could also be found in vines when selecting and moving towards the supports they want to attach to [17,18,19].

Finally, our current band analysis was conducted based on methods from neuroscience, but future studies should develop analyses tailored to the biology of plants. This will enable the uncovering of plant-specific electrophysiological patterns and bring to light the minutiae of the bioelectrical behaviour not only of *Cuscuta racemosa*, but other plant species as well. Furthermore, studies on plant attention are at their infancy, and more research must be conducted to corroborate this hypothesis. Once this phenomenon becomes clearer, we will have a better understanding of the evolution of a cognitive phenomenon that may be widespread beyond the animal kingdom. Indeed, selective attention seems to be critical to the survival of every organism. We should pay more attention to it.

## 4. Materials and Methods

All the methodology for acquiring the data was described in detail in [31]. Here, a summary of what was done is presented. The difference is in the electrophysiological analyses realised and described in Section 4.3.

### 4.1. Plant Material and Experimental Setup

Twigs of *Cuscuta racemosa* were collected from a stock grown on basil plants (*Ocimus* sp.) and kept in greenhouse conditions. They were trimmed so that every twig had a 10 cm length with only one node at one extremity, and their mean weight was 0.172 mg ± 0.042. They were placed inside a Styrofoam box (20 × 25 × 17 cm) which contained a shelf (12 × 17 cm) placed 5 cm deep inside the box. The shelf covered part of the box interior, which left a pit in the other half where the host plants were placed. Four dodders per box were placed in parallel with the longer side of the box with their nodes pointed to the pit. A pair of stainless-steel needle electrodes (model EL-452, BIOPAC Systems R^©^, Goleta, CA, USA) was inserted in the dodder’s stem immediately below the node and 1 cm apart from each other. The boxes were placed inside a Faraday cage and illuminated by a white LED light (PPFD 230 μmol m^−2^ s^−1^), with a photoperiod of 12.0 h and constant mean temperature of 25 °C ± 1. The boxes were sealed with transparent polyvinyl chloride (PVC) film to maintain humidity and then left in the laboratory overnight for acclimation.

### 4.2. Electrophysiological Recordings

In the following morning, the electrome of the dodders’ was recorded for 2 h before and 2 h after a pot with a host was introduced in the box. The introduced host was either a bean plant (*Phaseolus vulgaris* L. cv. BRS-Expedito) in the V3 stage of development, with the first trifoliolate leaf developing (following the classification of de Oliveira et al. [78]); or a wheat plant (*Triticum aestivum* L. cv. BRS-Parrudo), with the third leaf emerging after germination. This species was chosen as dodders cannot parasitise wheat plants and avoid them when possible [79,80,81].

The data was recorded with the system Biopac Student Lab (BIOPAC Systems R^©^, Goleta, CA, USA), model MP36 with four channels with a high input impedance (10 GΩ). Signals were collected with a sampling rate of fs = 62.5 Hz amplified with a gain of 1000-fold. The protocol used was ECG-AHA (0.05–100 Hz), with a notch frequency of 60.0 Hz to minimise the influence of the electrical network. The data obtained was a time series of micro-voltage variations as $\Delta V=\left\{\Delta V_{1},\;\Delta V_{2},\;\ldots ,\;\Delta V_{n}\right\}$, where $\Delta V_{i}$ is the difference of electrical potential between the electrodes, and n is the length of the time series. This length was derived from a sample of 2 h (7200 s) of data acquisition, and using fs = 62.5 Hz, *N* = 450,000 points. In the end, 23 time series for the test and 23 time series for the control were obtained and analysed. A detailed description of all the methods described until this point can be found in [31].

### 4.3. Electrophysiological Analyses

The fast Fourier transform (FFT) technique was used to understand the frequencies of the dodder’s electrome. For this work, we used Bluestein’s algorithm [82] and the rfft Hermitian-symmetric algorithm from scipy library [83] to silence imaginary values. The PSD (power spectral density) was calculated using the Welch method [84]. This method estimated the PSD by dividing the data in windows and calculating the periodogram for each window. Again, the scipy library was used [83] with the Welch method with 4 s windows. The PSD value returned was then used to calculate the ABP (average band power). This method consists of analysing the signals in specific frequencies. It is commonly used for the generation of EEG features in neuroscientific analyses [85,86,87,88,89,90,91], as well as in attention tests [92,93,94]. To calculate the ABP, the area determined by the interest must be integrated to the function provided by the PSD. This was done using the Simpson’s compound rule [95] which, in general, decomposes the area in many parabolas and then sum them up returning the value of the area of interest. For this calculation, the method Simpson from scipy library was used [83]. The band frequencies calculated were 0.0–0.5 Hz (here named low waves), 0.5–4.0 Hz (delta), 4.0–8 Hz (theta), 9–11 Hz (mu), 8–13 Hz (alpha), 13–30 Hz (beta), and 20–100 Hz (gamma), respectively.

To visualise these analyses, the method time dispersion analysis of features (TDAF) was employed [39]. To generate the characteristics, the time series were divided into shorter series, and with TDAF, the temporal information of the position of each clipping of the time series was kept and taken to each feature. After completing the calculations, it was possible to aggregate each characteristic for each stretch of time and make a dispersion analysis (with the maximum value, minimum value, median, and quartiles) in a specific moment. When all the analyses were plotted in the correct temporal order, it was then possible to obtain the dynamics of the dispersion of the feature for each time series analysed. Therefore, a qualitative way of analysing chaotic series through time was obtained.

## Data Availability

The raw data used in this work can be freely accessed in Parise et al. [31].
